# Associations of reproductive factors with postmenopausal follicle stimulating hormone

**DOI:** 10.1186/s40695-022-00079-6

**Published:** 2022-09-05

**Authors:** Rebecca Costa, Tomi-Pekka Tuomainen, Jyrki Virtanen, Leo Niskanen, Elizabeth Bertone-Johnson

**Affiliations:** 1 Department of Biostatistics and Epidemiology, University of Massachusetts, Amherst, MA USA; 2 Institute of Public Health and Clinical Nutrition, University of Eastern Finland, Kuopio, Finland; 3 Departments of Internal Medicine, Endocrinology/Diabetology, Päijät-Häme Central Hospital, Lahti, Finland; 4 Institute of Clinical Medicine, University of Eastern Finland, Kuopio, Finland; 5 Department of Health Promotion and Policy, University of Massachusetts, Amherst, MA USA; 6 Arnold House, University of Massachusetts, 715 North Pleasant Street, Amherst, MA 01003-9304 USA

**Keywords:** Follicle stimulating hormone, Postmenopause, Reproductive history, Hormone replacement therapy

## Abstract

**Purpose:**

Recent studies have suggested that higher postmenopausal follicle stimulating hormone (FSH) may be associated with lower risk of diabetes. However, relatively little is known about postmenopausal FSH levels, including the level of variation between women and whether reproductive factors are associated with this variation.

**Methods:**

We assessed the relationship of multiple reproductive factors with FSH levels among 588 postmenopausal women in the Kuopio Ischaemic Heart Disease Risk Factor Study. Participants were aged 53 to 73 years and not using hormone therapy at study enrollment (1998–2001) when reproductive factors were assessed and FSH was measured.

**Results:**

After adjustment for age, menopause timing, sex steroid levels, adiposity and behavioral factors, we observed numbers of pregnancies and age at first birth were each inversely associated with FSH levels. For example, women with ≥ 3 births and an age at first birth ≥ 25 years had mean FSH levels that were 7.8 IU/L lower than those of women with 1–2 births and an age at first birth ≤ 24 years (*P* = 0.003). Number of miscarriages was inversely associated with FSH levels (-2.7 IU/L per miscarriage; *P* = 0.02). Women reporting 4 or more years of past hormone therapy use had significantly higher mean FSH levels than women who had never used hormone therapy (*P* for trend = 0.006).

**Conclusion:**

Multiple reproductive factors were associated with postmenopausal FSH, independent of estradiol, adiposity and other confounders. These findings warrant replication and further exploration of potential underlying mechanism.

## Introduction

Recent studies have shown that postmenopausal follicle-stimulating hormone (FSH) may be inversely associated with prevalence of metabolic syndrome, diabetes and atherosclerosis in postmenopausal women [1–9]. Findings from these studies suggest that postmenopausal FSH may be predictive of future cardiovascular disease risk. However, little is known about postmenopausal FSH, including the level of variation between women and the factors associated with this variation. Given the important role of FSH in follicle development and ovulation in premenopausal women, we hypothesized that reproductive history may be associated with postmenopausal FSH.

FSH is involved in menstrual cycle regulation and fluctuates cyclically during the premenopausal years via feedback with estradiol. The release of FSH by the anterior pituitary stimulates the production of estradiol in the ovarian follicles. As the follicles mature, estrogen levels rise, creating a negative feedback effect with the anterior pituitary and causing FSH levels to decline. After estrogen levels decline at the end of the cycle, this negative feedback is broken, and the cycle repeats. As menopause approaches and follicular estradiol production declines, FSH levels increase in order to maintain estrogen levels, although the level of increase in FSH around the time of the final menstrual period varies among women [10]. Previous studies have suggested that there are distinct trajectories of FSH change during the menopausal transition, including low, medium, and high rising [10, 11]. At the conclusion of the menopausal transition, FSH levels are believed to plateau and gradually decline. However, few studies have directly evaluated FSH function in the years after the menopause transition is complete, though recent evidence suggests that postmenopausal FSH levels between women may vary substantially, independent of estrogens and adiposity [1, 11].

Few studies have assessed whether reproductive factors including parity, age at first birth, age at menarche, and exogenous hormone use may be associated with postmenopausal FSH levels [12, 13]. We evaluated the relation of multiple reproductive factors and postmenopausal FSH while accounting for other factors including adiposity, hormones, and behavioral factors among a population of Finnish women.

## Methods

### Study population

The current analysis uses baseline data from the Kuopio Ischaemic Heart Disease Risk Factor Study (KIHD), an ongoing population-based cohort study of cardiovascular and metabolic health in men and women in eastern Finland. Female participants were first enrolled between March 1998 and February 2001, and included a random sample of 1173 postmenopausal women living in and around Kuopio. Women were selected from four specific age groups that reflected the age distribution of the male cohort: 53–56 years, 59–62 years, 64–68 years, and 71–73 years. The cohort was ultimately comprised of 920 women (78.4% of eligible women) who completed the baseline clinical assessments. Of those not participating, 168 refused, 51 were unreachable, and 34 were not included for other reasons. The study protocol was approved by the Research Ethics Committee of the University of Kuopio. All participants provided written informed consent.

### Assessments of reproductive factors and clinical factors

Participants provided data on demographic, behavior, reproductive, and health-related factors on study questionnaires, which were reviewed for completeness and clarity by a trained interviewer. Reproductive factors included age at menarche, history and duration of oral contraceptive use, number of full-term pregnancies, age at last menses, and history of hysterectomy and oophorectomy; use of hormone therapy (HT; ever use, current use and total duration) was also assessed. Postmenopausal status and age at menopause were defined by the absence of menses for at least 12 months, or at the time of oophorectomy for women who reported having undergone surgery prior to menopause. The KIHD 12-Month Leisure Time Physical Activity History questionnaire was used to measure recreational activity [14] and estimate metabolic equivalent of task hours per day. Each participant also completed a detailed alcohol use questionnaire [15]. Height and weight were directly measured and used to calculate BMI (weight in kg/square of height in m). Waist and hip circumferences were measured with a standard measuring tape and used to calculate waist-to-hip ratio.

### Blood collection and assessment of FSH

Fasting blood samples were collected between 8:00 AM and 10:00 AM during the clinical interview, after participants had abstained from eating or smoking for 12 h and drinking alcohol for 3 days. Plasma was separated from other blood components within 60 min and stored at − 20 °C or − 80 °C until assay. Samples were assayed for FSH between June 2001 and February 2002. Serum FSH concentration was determined with a sandwich technique applying an immunoradiometric assay manufactured by Diagnostic Products Corporation (Coat-A-Count FSH IRMA; Diagnostic Products Corporation, Los Angeles, California). Serum 17-B-estradiol was assayed between 1999 and 2001 with a radioimmunoassay manufactured by DiaSorin (DiaSorin S.p.A., Stillwater, Minnesota); assay sensitivity was 18.4 pmol/L [16. Serum testosterone (17B-hydroxy-4-androsten-3-one) was determined with the Spectria Testosterone radioimmunoassay kit (Orion Diagnostic, Espoo, Finland); assay sensitivity was 0.10 nmol.L [16]. I label measurements for FSH, E_2_, and testosterone were carried out by gamma counter Wallac 1261 MultiGamma using a RiaCalc LM Evaluation Program. Inter-assay coefficients of variation (CVs) were 5% for FSH, 7.6% to 12.0% for E_2_, and 7.9% to 12.2% for testosterone. Sex hormone binding globulin (SHBG) was assayed using the 1235 AutoDELFIA automatic assay system (PerkinsElmer Wallac Oy, Turku, Finland) based on a time-resolved fluoroimmunoassay; Inter-assay CVs ranged from 6.0–9.0%.

### Statistical analysis

The present analysis was limited to women not using HT at baseline (n = 593). Additionally, five participants did not have FSH measurements and were excluded, leaving 588 women as the analytic population. FSH was normally distributed and did not require transformation. We divided the participants by age based on the categories used for participant recruitment (53–56, 59–62, 64–68, 71–73 years) and compared mean FSH levels between the four categories using analysis of variance.

To determine if participant characteristics were related to FSH levels, we evaluated these associations using linear regression. We then used linear regression to assess how reproductive factors, including number of full-term births, timing of first pregnancy, history of spontaneous abortion, age at menarche, age at menopause, number of reproductive years, OC use and duration, and past HT use and duration were associated with FSH. Indicator variables were created for fixed categories. To account for confounding by demographic, hormonal and behavioral factors, we built two multivariate models. In the first, we adjusted for year of study entry, age, sex steroid hormone levels (estradiol, testosterone, and SHBG), adiposity (BMI and waist to hip ratio), and behavioral factors (current smoking, pack-years of smoking, physical activity, and alcohol use (g/week). In the second model, we adjusted reproductive factors for each other. Information on variable categorizations is included in table footnotes.

## Results

Mean FSH levels did not vary significantly across age groups (Table 1; *P* = 0.09). The standard deviation was slightly higher for women 53–56, but was similar across other age groups, indicating that variation was substantial and similar in magnitude among women in their late 50 s to early 70 s.

**Table 1:** Distribution of postmenopausal follicle-stimulating hormone levels among *n* = 588 women not currently using hormone therapy, by age group, Kuopio Ischaemic Heart Disease Risk Factor Study, 1998–2001

		Follicle-Stimulating Hormone Levels (IU/L)					
Age	n	Mean	SD	Median	IQR	Min	Max
53–56	114	54.7	25.3	31.8	31.8	1.5	136.8
59–62	130	51.6	18.8	20.9	20.9	2.4	108.6
64–68	163	51.3	16	17	17	4.8	102.8
71–73	181	48.9	17	23.2	23.2	3.6	99.1

As shown in Table 2, FSH levels were inversely associated with age and BMI, but were not associated with physical activity, alcohol intake, and smoking. For example, each 1-year increase in age was associated with a -0.29 IU/L decrease in mean FSH level (P = 0.02). FSH was significantly inversely associated with estradiol, and testosterone, and significantly positively associated with SHBG.

**Table 2:** Beta-coefficients for the association of demographic, behavior and hormonal factors with follicle-stimulating hormone levels among n = 588 postmenopausal women aged 53–73 who were not using hormone therapy. Kuopio Ischaemic Heart Disease Risk Factor Study, 1998–2001

	Follicle-stimulating hormone (IU/L)	
Characteristic	Beta (SE)^a^	P
Age (year)	-0.29 (0.12)	0.02
Body mass index (kg/m^2^)	-1.03 (0.14)	< 0.0001
Physical activity (MET h/d)	0.20 (0.12)	0.09
Alcohol intake (g/wk)	-0.02 (0.02)	0.31
Smoking (pack-years)^b^	-0.07 (0.12)	0.57
Estradiol (pmol/L)	-0.13 (0.02)	< 0.0001
Testosterone (nmol/L)	-2.01 (0.51)	< 0.0001
Sex Hormone Binding Globulin (nmol/L)	0.16 (0.03)	< 0.0001

Abbreviationss: *MET* metabolic equivalent of task

^a^Beta represents change in mean FSH level (IU/L) per one-unit increase in level of each characteristic

^b^Pack-years of smoking among ever smokers

Association of pregnancy-related reproductive factors with FSH levels are presented in Table 3. In unadjusted analyses, higher parity and older age at first birth were each associated with lower FSH levels. Associations were somewhat attenuated after adjustment for demographic, hormonal and behavioral factors (MV1). In fully-adjusted models (MV2), parity was significantly and inversely associated with FSH (P for trend = 0.03). In the analysis of both factors simultaneously, women with ≥ 3 births and an age at first birth ≥ 25 years had mean FSH levels that were 7.8 IU/L lower than those of women with 1–2 births and an age at first birth ≤ 24 years (*P* = 0.003).

**Table 3:** Beta-coefficients for the association of pregnancy-related factors with follicle-stimulating hormone levels among n = 588 postmenopausal women aged 53–73 who were not using hormone therapy. Kuopio Ischaemic Heart Disease Risk Factor Study, 1998–2001

		Unadjusted		Multivariable Model 1^a^		Multivariable Model 2^b^	
Pregnancy-related characteristic	n	beta (SE)	P	beta (SE)	P	beta (SE)	P
Number of full-term pregnancies							
0	76	1.9 (2.6)	0.47	2.5 (2.4)	0.30	2.2 (2.5)	0.39
1	94	0.7 (2.4)	0.79	2.1 (2.2)	0.35	2.9 (2.3)	0.21
2	175	referent		referent		referent	
3	128	-5.5 (2.2)	0.01	-4.0 (2.0)	0.05	-3.6 (2.1)	0.08
≥ 4	128	-3.3 (2.3)	0.16	-0.8 (2.2)	0.71	-1.6 (2.3)	0.48
per pregnancy		-1.2 (0.5)	0.009	-0.9 (0.4)	0.04	-1.0 (0.5)	0.03
Age at first birth (years)							
< 20	50	0.5 (3.0)	0.86	-0.9 (2.7)	0.75	-1.1 (2.7)	0.70
20—24	253	referent		referent		referent	
25—29	145	-2.5 (2.0)	0.21	-1.9 (1.8)	0.29	-2.1 (1.8)	0.26
≥ 30	59	-4.9 (2.8)	0.08	-3.4 (2.5)	0.17	-3.8 (2.6)	0.15
per 1 year		-0.3 (0.2)	0.07	-0.2 (0.2)	0.18	-0.3 (0.2)	0.16
Cross-classification of pregnancies by age at first birth							
0	76	-1.2 (2.7)	0.65	-0.1 (2.5)	0.96	-0.9 (2.6)	0.72
1–2 pregnancies, < 25 years old	132	referent		referent		referent	
3 + pregnancies, < 25 years old	171	-6.4 (2.2)	0.004	-3.8 (2.0)	0.06	-4.4 (2.1)	0.03
1–2 pregnancies, ≥ 25 years old	135	-5.4 (2.3)	0.02	-2.8 (2.1)	0.18	-3.3 (2.2)	0.13
3 + pregnancies, ≥ 25 years old	69	-9.7 (2.8)	0.0006	-7.3 (2.6)	0.01	-7.8 (2.6)	0.003
Number of miscarriages^c^		-2.7 (1.2)	0.03	-2.9 (1.1)	0.01	-2.7 (1.2)	0.02
Number of induced abortions^c^		0.9 (1.9)	0.63	-0.7 (1.7)	0.68	-0.01 (1.9)	0.995

^a^Adjusted for year of enrollment, age (years), estradiol (pmol/L), testosterone (nmol/L), and sex hormone binding globulin (nmol/L)

^b^Adjusted for covariates in MV 1 and body mass index (continuous, kg/m^2^), waist-to-hip ratio (quartiles), physical activity (MET hours/day), alcohol intake (g/week), currents smoking (yes, no), pack-years of smoking (continuous), duration of OC use (categorical) and duration of past HT use (categorical)

^c^beta per each spontaneous or induced abortion

The reported number of miscarriages among participants ranged from 0 to 5, with *n* = 104 women reporting 1 and *n* = 30 reporting 2 or more miscarriages. Number of miscarriages was inversely associated with FSH level. In our fully adjusted model, each miscarriage was associated with a 2.7 IU/L lower FSH level.

As shown in Table 4, neither age at menarche or at last menses were associated with FSH level. Duration of OC use was not consistently associated with FSH. In our fully adjusted model (model 3), mean FSH levels were 0.3–7.5 IU/L higher in women reporting past HT for 4–6 years or 7 + years, compared to those who had never used HT.

**Table 4:** Beta-coefficients for the association of menstrual factors and exogenous hormone use with follicle-stimulating hormone levels among n = 588 postmenopausal women aged 53–73 who were not using hormone therapy. Kuopio Ischaemic Heart Disease Risk Factor Study, 1998–2001

		Unadjusted		MV 1		MV2	
Characteristic	n	beta (SE)	P	beta (SE)	P	beta (SE)	P
Age at menarche (years)							
< 12	105	-0.2 (2.5)	0.94	1.4 (2.3)	0.52	3.3 (2.4)	0.16
13	140	referent		referent		referent	
14	128	-0.3 (2.3)	0.89	-0.1 (2.1)	0.97	-0.1 (2.3)	0.97
15	113	-1.2 (2.4)	0.62	-0.3 (2.2)	0.89	1.6 (2.4)	0.50
≥ 16	94	1.9 (2.6)	0.45	2.0 (2.3)	0.39	1.6 (2.4)	0.53
per 1 year		0.3 (0.5)		0.1 (0.5)	0.89	-0.2 (0.5)	0.66
Age at last menses (years)							
< 50	257	2.6 (1.8)	0.15	1.5 (1.6)	0.35	0.6 (1.7)	0.74
50 – 52	204	referent		referent		referent	
> 52	127	-0.4 (2.2)	0.85	-0.3 (2.0)	0.87	-0.1 (2.1)	0.97
per 1 year		-0.2 (0.2)	0.40	-0.1 (0.2)	0.73	0.1 (0.2)	0.74
Duration of oral contraceptive use (years)							
None	398	referent		referent		referent	
< 1	38	1.4 (3.2)	0.67	0.9 (3.1)	0.78	0.1 (3.2)	0.97
1 – 3	65	0.9 (2.6)	0.71	2.4 (2.4)	0.32	2.4 (2.5)	0.32
4 – 6	41	-1.3 (3.1)	0.69	-1.9 (2.9)	0.50	-1.3 (2.9)	0.64
≥ 7	35	8.9 (3.4)	0.01	5.4 (3.1)	0.08	5.6 (3.2)	0.08
Test for trend			0.04		0.23		0.18
Duration of hormone therapy use^c^ (years)							
None	393	referent		referent		referent	
< 1	56	-0.9 (2.7)	0.75	-1.3 (2.5)	0.60	0.2 (2.5)	0.96
1 – 3	59	1.0 (2.7)	0.70	-1.9 (2.4)	0.43	-0.6 (2.6)	0.83
4 – 6	31	8.2 (3.6)	0.02	8.1 (3.2)	0.01	7.3 (3.4)	0.03
≥ 7	33	7.3 (3.5)	0.04	7.4 (3.2)	0.02	7.5 (3.2)	0.03
Test for trend			0.005		0.003		0.006

^a^Adjusted for year of enrollment, age (years), estradiol (pmol/L), testosterone (nmol/L), and sex hormone binding globulin (nmol/L)

^b^Adjusted for covariates in MV 1 and body mass index (continuous, kg/m^2^), waist-to-hip ratio (quartiles), physical activity (MET hours/day), alcohol intake (g/week), currents smoking (yes, no), pack-years of smoking (continuous), parity (continuous), age at first birth (continuous), number of miscarriages (continuous)

^c^Previous HT use; all participants included were not using HT at the time of FSH measurement

## Discussion

In this study of older postmenopausal women, we observed significant associations between several reproductive factors and FSH levels. Higher parity, later age at first birth and number of miscarriages were inversely associated with FSH, while longer duration of past HT use was positively associated with levels. Importantly, these associations existed after adjustment for sex steroids, adiposity measures, behavioral factors and the other reproductive factors assessed.

To our knowledge, an association between reproductive factors and FSH, accounting for other factors including adiposity, hormones, and behavioral factors, has not been observed before. While two previous studies of reproductive factors and FSH levels suggested potential associations of pregnancy history, neither study accounted for potential confounding by sex steroid hormone levels. Consistent with our results showing lower mean FSH levels among parous women, Chubak et al. found that among 173 postmenopausal, sedentary, overweight or obese women, nulliparous women had 19% higher FSH concentrations than parous women (*p* = 0.02), even after adjustment for age, body fat, alcohol consumption, marital status, race, and number of ovaries remaining [12]. Ness et al. reported that among 270 postmenopausal women, pregnancy termination was significantly related to a rise in FSH one year after menopause (*p* = 0.03), after adjustment for BMI, education, oral contraceptive use, smoking, and alcohol consumption [13]. However, this study focused on younger, as opposed to older, postmenopausal women. Consistent with our results, both of these studies found no association between age at menarche or at last menses and FSH levels.

The associations we observed between reproductive factors and FSH levels could plausibly be explained by confounding by adiposity, as higher adiposity is associated with higher endogenous estrogen production and consequently lower FSH levels. However, since we adjusted for both BMI and waist to hip ratio, confounding by adiposity and associations of adiposity with estrogens is unlikely to explain our findings.

These results lend support for the hypothesis that actions of FSH may in part underlie observed relations of reproductive factors with diabetes. Emerging evidence suggests that in postmenopausal women, high FSH levels may be associated with lower risk of insulin resistance, metabolic syndrome and T2D [1, 3–9]. In a previous analysis in the KIHD population, we found that each 1 IU/L increase in FSH was associated with a 1.9% lower prevalence of diabetes at baseline [1]. Furthermore, women with FSH levels above the median (50 IU/L) had approximately half the risk of developing T2D over 7 to 9 years of follow-up than women with lower levels. Wang et al. found that participants with FSH levels of 50.2 IU/L or less had three times higher risk of prevalent diabetes than women with FSH levels of at least 82.5 IU/L [3]. Bjørnerem et al. found that women with diabetes had geometric mean postmenopausal FSH levels that were 7.4 IU/L lower than levels healthy postmenopausal women (*P* = 0.03) [4].

Recent laboratory studies have reported the presence of FSH receptors and actions of FSH in tissues beyond the reproductive track. For example, in islet cells from the rat pancreas, Chu et al. observed that FSH receptor expression and insulin and glucagon secretion were altered by FSH [17]. A growing number of observational studies have observed inverse associations of postmenopausal FSH levels with insulin and/or fasting glucose levels [1, 5, 6].

Multiple previous studies have observed that higher parity is associated with an increased risk of T2D, which persists after menopause [18–25]. For example, in the Singapore Chinese Health Study of women 45–74 at baseline [18], number of live births was linearly associated with incidence of diabetes over an average follow-up of 5.7 years; compared to nulliparous women, those with 3–4 and > 5 births had 62% and 74% higher risk, respectively (*P* for trend < 0.001). Our observations suggest that actions of FSH may, to some extent, underlie these relations.

Our finding that longer past HT use is associated with higher FSH level is somewhat unexpected. As exogenous estrogen use stimulates negative feedback and tends to lower gonadotropin levels [26], we hypothesized that past HT use could contribute to lower FSH. These finding warrants replication in prospective studies.

Multiple reproductive hormones likely contribute to relations between reproductive factors and diabetes risk, including estradiol and SHBG. In our population, postmenopausal FSH is inversely associated with estradiol and testosterone, and positively associated with SHBG. It is important to note that our multivariable models adjust for these hormones; consequently, findings regarding the relation of FSH and reproductive factors should be unconfounded by these factors. However, we suggest that future study of the relation of FSH and diabetes risk should specifically address potential confounding or mediation by SHBG and estradiol.

Our study has several limitations. We are unable to evaluate the extent to which postmenopausal FSH levels vary within women, as our classification was based on a single assessment. While the use of a single sample may lead to misclassification, we expect this to be unrelated to reproductive history and thus not an explanation for the associations we observed. It is worth noting the possibility that reproductive history and postmenopausal FSH may both be influenced by factors relating to ovarian function during the premenopausal years. Prospective studies with repeated measures of FSH across the life span are needed to fully understand these relations.

KIHD participants are all residents of eastern Finland and quite similar in terms of race/ethnicity and cultural factors; we recommend that additional studies in diverse populations be conducted to determine if findings are replicated. We were able to adjust for a large number of confounders in our analysis, and found that associations persisted after adjustment, suggesting that associations for FSH are not explained by these factors or by other sex steroids. There may be residual confounding if some confounders are misclassified. As our participants are 53 and older, some of the historical data may be recalled incorrectly and some current data may be inaccurately reported. Fortunately, as estradiol, testosterone, SHBG, and BMI were directly measured, confounding by these important variables is less likely. Some residual confounding in sex steroids may be caused by measurement error in immunoassays, which may not be sufficiently sensitive to precisely measure very low levels. However, the particular assays used in our study have demonstrated reasonable validity in postmenopausal women [16].

To our knowledge, this is among the first studies of reproductive factors and postmenopausal FSH levels. We observed evidence of significantly lower FSH levels among women with high parity, later age at first birth, and miscarriage, and significant higher FSH levels among women with a long duration of HT use. These associations were not explained by estradiol, adiposity, and other factors. Additional studies of reproductive factors and FSH in older postmenopausal women are needed to explore potential underlying physiological explanations, and whether FSH also underlies associations of reproductive factors with diabetes risk.

## Data Availability

The data used in this study are available from the KIHD study, but restrictions apply to their availability. Data are available from the authors upon reasonable request and with permission of the KIHD study.

## References

[1] Bertone-Johnson ER, Virtanen JK, Niskanen L (2017). Association of follicle-stimulating hormone levels and risk of type 2 diabetes in older postmenopausal women. Menopause.

[2] Bertone-Johnson ER, Virtanen JK, Nurmi T (2018). Follicle-Stimulating Hormone Levels and Subclinical Atherosclerosis in Older Postmenopausal Women. Am J Epidemiol.

[3] Wang N, Kuang L, Han B (2016). Follicle-stimulating hormone associates with prediabetes and diabetes in postmenopausal women. Acta Diabetol.

[4] Bjørnerem A, Straume B, Midtby M (2004). Endogenous Sex Hormones in Relation to Age, Sex, Lifestyle Factors, and Chronic Diseases in a General Population: The Tromsø Study. J Clin Endocrinol Metab.

[5] Güdücü N, Görmüş U, Kutay SS, Kavak ZN, Telatar B (2013). Endogenous sex hormones and their associations with cardiovascular risk factors in post-menopausal women. J Endocrinol Invest.

[6] Stefanska A, Ponikowska I, Cwiklinska-Jurkowska M, Sypniewska G (2014). Association of FSH with metabolic syndrome in postmenopausal women: a comparison with CRP, adiponectin and leptin. Biomarkers Med.

[7] Stefanska A, Sypniewska G, Ponikowska I, Cwiklinska-Jurkowska M (2012). Association of follicle-stimulating hormone and sex hormone binding globulin with the metabolic syndrome in postmenopausal women. Clin Biochem.

[8] Stefanska A, Cembrowska P, Kubacka J, Kuligowska-Prusinska M, Sypniewska G (2019). Gonadotropins and their association with the risk of prediabetes and type 2 diabetes in middle-aged postmenopausal women. Dis Markers.

[9] Jung ES, Choi EK, Park BH, Chae SW (2020). Serum follicle-stimulating hormone levels are associated with cardiometabolic risk factors in post-menopausal Korean women. J Clin Med.

[10] Tepper PG, Randolph JF, McConnell DS (2012). Trajectory clustering of estradiol and follicle-stimulating hormone during the menopausal transition among women in the Study of Women’s Health across the Nation (SWAN). J Clin Endocrinol Metab.

[11] Yasui T, Ideno Y, Onizuka Y, Nakajima-Shimada J, Shinozaki H, Hayashi K (2019). Variation of urinary follicle-stimulating hormone level after menopause: From the results of the Japan Nurses’ Health Study. J Med Invest.

[12] Chubak J, Tworoger SS, Yasui Y, Ulrich CM, Stanczyk FZ, McTiernan A (2004). Associations between Reproductive and Menstrual Factors and Postmenopausal Sex Hormone Concentrations. Cancer Epidemiol Biomarkers Prev.

[13] Ness RB, Buhari A, Gutai J, Kuller LH (2000). Reproductive history in relation to plasma hormone levels in healthy post-menopausal women. Maturitas.

[14] Lakka TA, Venäläubeb JM, Rauramaa R (1994). Relation of leisure-time physical activity and cardiorespiratory fitness to risk of acute myocardial infarction. N Eng J Med.

[15] Voutilainen S, Rissanen TH, Virtanen J (2001). Low dietary folate is associated with an excess incidence of acute coronary events: The Kuopio Ischemic Heart Disease Risk Factor Study. Circulation.

[16] Rinaldi S, Dechaud H, Biessy C (2001). Reliability and validity of commercially available, direct radioimmunoassays for measurement of blood androgens and estrogens in postmenopausal women. Cancer Epidemiol Biomarkers Prev. Cancer Epidemiol Biomarkers Prev.

[17] Chu C, Xu B, Huang W (2010). A study on expression of FSH and its effects on the secretion of insulin and glucagon in rat pancreas. Tissue Cell.

[18] Mueller NT, Mueller NJ, Odegaard AO (2013). Higher parity is associated with an increased risk of type-II diabetes in Chinese women: the Singapore Chinese Health Study. BJOG.

[19] Luo J, Hendryx M, LeBlanc ES (2019). Associations Between Parity, Breastfeeding, and Risk of Maternal Type 2 Diabetes Among Postmenopausal Women. Obstet Gynecol.

[20] Tian Y, Shen L, Wu J (2014). Parity and the risk of diabetes mellitus among Chinese women: a cross-sectional evidence from the Tongji-Dongfeng cohort study. PLoS ONE.

[21] Araneta MRG, Barrett-Connor E (2010). Grand multiparity is associated with type 2 diabetes in Filipino American women, independent of visceral fat and adiponectin. Diabetes Care.

[22] Naver KV, Lundbye-Christensen S, Gorst-Rasmussen A (2011). Parity and risk of diabetes in a Danish nationwide birth cohort. Diabet Med.

[23] Nicholson WK, Asao K, Brancati F, Coresh J, Pankow JS, Powe NR (2006). Parity and Risk of Type 2 Diabetes. Diabetes Care.

[24] Cure P, Hoffman HJ, Cure-Cure C (2015). Parity and diabetes risk among hispanic women from Colombia: cross-sectional evidence. Diabetol Metab Syndr.

[25] Kirtz-Silverstein D, Barrett-Connor E, Wingard DL (1989). The effect of parity on the later development of non-insulin-dependent diabetes mellitis or impaired glucose tolerance. New Engl J Med.

[26] King JM, Dowling NM, Bimonte-Nelson HA (2019). Impact of menopausal hormone formulations on pituitary-ovarian regulatory feedback. Am J Physiol Regul Integr Comp Physiol.

